# 2,2′-(Propane-2,2-di­yl)dibenzothia­zole

**DOI:** 10.1107/S1600536810035488

**Published:** 2010-09-08

**Authors:** Spring Melody M. Knapp, Lev N. Zakharov, David R. Tyler

**Affiliations:** a Department of Chemistry, 1253 University of Oregon, Eugene, Oregon 97403-1253, USA

## Abstract

The two symmetry-independent mol­ecules in the asymmetric unit of the title compound, C_17_H_14_N_2_S_2_, have similar geometry; the dihedral angles between the least-squares planes of the benzothia­zole groups in the two mol­ecules are 83.93 (3) and 81.26 (3)°.

## Related literature

For the synthesis of similar compounds, see: Avendaño *et al.* (1988 ▶); Kelarev *et al.* (2003 ▶); Babudri *et al.* (1986 ▶). For literature regarding nitrile hydration, see: Ahmed *et al.* (2009 ▶). For results on nitrile hydratase, see: Nagasawa & Yamada (1989 ▶); Kobayashi *et al.* (1992 ▶). For nitrile hydratase mimics, see: Noveron *et al.* (2001 ▶); Tyler *et al.* (2003 ▶); Yano *et al.* (2008 ▶).
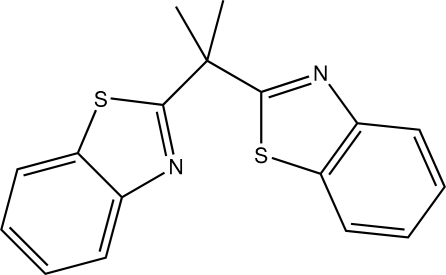


## Experimental

### 

#### Crystal data

C_17_H_14_N_2_S_2_
*M* *_r_* = 310.42
Triclinic, 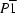

*a* = 10.3791 (13) Å
*b* = 11.8832 (15) Å
*c* = 12.3391 (15) Å
α = 86.730 (2)°
β = 78.048 (2)°
γ = 80.779 (2)°
*V* = 1469.2 (3) Å^3^
*Z* = 4
Mo *K*α radiation
μ = 0.36 mm^−1^
*T* = 173 K
0.42 × 0.24 × 0.05 mm

#### Data collection

Bruker APEX CCD area-detector diffractometer
Absorption correction: multi-scan (*SADABS*; Sheldrick, 1995 ▶) *T*_min_ = 0.865, *T*_max_ = 0.982
17048 measured reflections
6379 independent reflections
5007 reflections with *I* > 2σ(*I*)
*R*_int_ = 0.032

#### Refinement

*R*[*F* ^2^ > 2σ(*F* ^2^)] = 0.045
*wR*(*F* ^2^) = 0.119
*S* = 1.04
6379 reflections
491 parameters
All H-atom parameters refined
Δρ_max_ = 0.43 e Å^−3^
Δρ_min_ = −0.21 e Å^−3^

### 

Data collection: *SMART* (Bruker, 2000 ▶); cell refinement: *SAINT* (Bruker, 2000 ▶); data reduction: *SAINT*; program(s) used to solve structure: *SHELXTL* (Sheldrick, 2008 ▶); program(s) used to refine structure: *SHELXTL*; molecular graphics: *SHELXTL*; software used to prepare material for publication: *SHELXTL*.

## Supplementary Material

Crystal structure: contains datablocks I, global. DOI: 10.1107/S1600536810035488/ya2128sup1.cif

Structure factors: contains datablocks I. DOI: 10.1107/S1600536810035488/ya2128Isup2.hkl

Additional supplementary materials:  crystallographic information; 3D view; checkCIF report

## Figures and Tables

**Fig. 1.**
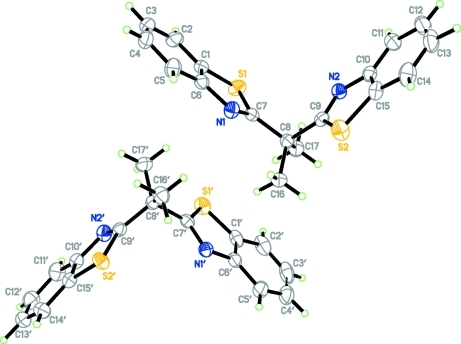
Asymmetric unit of the structure of the title compound with displacement ellipsoids, drawn at 50% probability level.

## supplementary crystallographic information

### Comment

Investigations in our lab are focused on the synthesis and study of nitrile hydration catalysts and their activity with respect to cyanohydrin substrates (Ahmed *et al.*, 2009). Catalysts investigated by our group have been found to be susceptible to poisoning by cyanide produced from cyanohydrin decomposition. Nitrile hydratase enzymes are capable of hydrating cyanohydrins (Nagasawa & Yamada, 1989; Kobayashi *et al.*, 1992). Additionally, some nitrile hydratase mimics have successfully hydrated acetonitrile in the presence of free cyanide (Tyler *et al.*, 2003). With the goal of investigating these mimics for their activity towards cyanohydrins, attempts were made to synthesize nitrile hydratase mimics similar to those made earlier by Tyler *et al.* (2003), but based on dimethylmalonyl dichloride, rather than 2,6-pyridinedicarbonyl dichloride.

The present X-ray study of the product showed that deprotection of 2,2-dimethyl-*N*,*N*'-bis(2-(tritylthio)phenyl)malonamide with trifluoroacetic acid and triethylsilane resulted in a ring closing condensation which yielded 2,2-bis(benzothiazole)propane, rather than the desired *N*,*N*'-bis(2-mercaptophenyl)-2,2-dimethylmalonamide.

There are two symmetry independent, but geometrically very similar molecules in the crystal of the title compound (Fig. 1). Dihedral angles between the least squares planes of the benzothiazole groups are equal to 83.93 (3) and 81.26 (3)° in molecules N1—C17 and N1'-C17', respectively.

### Experimental

The title compound was synthesized in three steps. Tritylated aminothiophenol was prepared following the literature procedure (Noveron *et al.*, 2001). Under a nitrogen atmosphere, dimethylmalonyl dichloride (0.40 ml, 2.97 mmol) was dissolved in a solution of triethylamine (0.95 g, 6.84 mmol) in 10 ml of chloroform, then added dropwise to a degassed solution of tritylated aminothiophenol (2.190 g, 5.95 mmol) and triethylamine (0.95 g, 6.84 mmol) in 10 ml of chloroform. The mixture was allowed to react for 16 h at room temperature, and then the solvent was removed under vacuum. Cold methanol was added to the tan solid, then filtered under vacuum. The precipitate was washed with cold methanol to yield a colorless solid, 2,2-dimethyl-*N*,*N*'-bis(2-(tritylthio)phenyl)malonamide (Try-DMPS). Yield: 6.02 g (71%). ^1^H NMR (CDCl_3_, 300 MHz) δ from TMS: 1.17 (s, 6H), 6.84 (t, 2H), 7.19–7.34 (m, 34H), 8.4 (d, 2H), 9.22 (s, 2H). ^13^C NMR (CDCl_3_, 75.4 MHz) δ 24.2, 51.7, 71.9, 120.3, 122.1, 123.7, 127.3, 127.9, 130.2, 131.1, 137.1, 142.5, 143.9, 170.9. Selected IR bands (KBr pellet, cm^-1^) 3348 (w), 3056 (w), 1688 (m), 1575 (m), 1504 (s), 1430 (m), 1297 (m), 701 (s). Try-DMPS (1.45 g, 1.75 mmol) was added to a mixture of 5 mL of trifluoroacetic acid and 3 mL of dichloromethane under stirring, and the solution instantly turned bright red. Triethylsilane (0.56 ml, 3.5 mmol) was added dropwise, with the color gradually changing from red to yellow to colorless. The solution was stirred for 15 minutes at room temperature, and then dichloromethane was removed under vacuum. The slurry was filtered to remove the triphenylmethane sideproduct, and the solvent was removed from the filtrate under vacuum to obtain a colorless solid. The solid was recrystallized twice in methanol to yield X-ray quality colorless crystals of the title compound. Yield: 1.4504 g (43%). ^1^H NMR (CDCl_3_, 300 MHz) δ from TMS: 2.18 (s, 6H), 7.36 (t, 2H), 7.41 (t, 2H), 7.85 (dd, 2H), 8.07 (d, 2H). ^13^C NMR (CDCl_3_, 75.4 MHz) δ 29.7, 47.8, 121.8, 123.5, 125.4, 126.3, 135.8, 153.1, 176.9. Selected IR bands (KBr pellet, cm^-1^) 3427 (w), 2986 (m), 1492 (s), 1435 (s), 1207 (s), 760 (s).

### Refinement

The H atoms were located in the difference map and included in the subsequent refinement with isotropic thermal parameters; C—H 0.88 (3)–1.04 (2) Å.

### Crystal data

**Table:**

C_17_H_14_N_2_S_2_	*Z* = 4
*M**_r_* = 310.42	*F*(000) = 648
Triclinic, *P*1	*D*_x_ = 1.403 Mg m^−^^3^
Hall symbol: -P 1	Mo *K*α radiation, λ = 0.71073 Å
*a* = 10.3791 (13) Å	Cell parameters from 3754 reflections
*b* = 11.8832 (15) Å	θ = 2.4–26.4°
*c* = 12.3391 (15) Å	µ = 0.36 mm^−^^1^
α = 86.730 (2)°	*T* = 173 K
β = 78.048 (2)°	Plate, colorless
γ = 80.779 (2)°	0.42 × 0.24 × 0.05 mm
*V* = 1469.2 (3) Å^3^	

### Data collection

**Table:**

Bruker APEX CCD area-detector diffractometer	6379 independent reflections
Radiation source: fine-focus sealed tube	5007 reflections with *I* > 2σ(*I*)
graphite	*R*_int_ = 0.032
phi and ω scans	θ_max_ = 27.0°, θ_min_ = 1.7°
Absorption correction: multi-scan (*SADABS*; Sheldrick, 1995)	*h* = −13→13
*T*_min_ = 0.865, *T*_max_ = 0.982	*k* = −15→15
17048 measured reflections	*l* = −15→15

### Refinement

**Table:**

Refinement on *F*^2^	Primary atom site location: structure-invariant direct methods
Least-squares matrix: full	Secondary atom site location: difference Fourier map
*R*[*F*^2^ > 2σ(*F*^2^)] = 0.045	Hydrogen site location: difference Fourier map
*wR*(*F*^2^) = 0.119	All H-atom parameters refined
*S* = 1.04	*w* = 1/[σ^2^(*F*_o_^2^) + (0.0637*P*)^2^ + 0.1616*P*] where *P* = (*F*_o_^2^ + 2*F*_c_^2^)/3
6379 reflections	(Δ/σ)_max_ = 0.001
491 parameters	Δρ_max_ = 0.43 e Å^−^^3^
0 restraints	Δρ_min_ = −0.21 e Å^−^^3^

### Special details

**Table:**

Geometry. All e.s.d.'s (except the e.s.d. in the dihedral angle between two l.s. planes)are estimated using the full covariance matrix. The cell e.s.d.'s are takeninto account individually in the estimation of e.s.d.'s in distances, anglesand torsion angles; correlations between e.s.d.'s in cell parameters are onlyused when they are defined by crystal symmetry. An approximate (isotropic)treatment of cell e.s.d.'s is used for estimating e.s.d.'s involving l.s. planes.
Refinement. Refinement of *F*^2^ against ALL reflections. The weighted *R*-factor *wR* and goodness of fit *S* are based on *F*^2^, conventional *R*-factors *R* are based on *F*, with *F* set to zero for negative *F*^2^. The threshold expression of *F*^2^ > σ(*F*^2^) is used only for calculating *R*-factors(gt) *etc*. and is not relevant to the choice of reflections for refinement. *R*-factors based on *F*^2^ are statistically about twice as large as those based on *F*, and *R*- factors based on ALL data will be even larger.

### Fractional atomic coordinates and isotropic or equivalent isotropic displacement parameters (Å^2^)

**Table:**

	*x*	*y*	*z*	*U*_iso_*/*U*_eq_	
S1	0.11702 (5)	0.06830 (4)	0.74433 (5)	0.03295 (15)	
S2	0.15867 (6)	0.39956 (5)	0.46027 (5)	0.03744 (16)	
N1	0.07371 (17)	0.28776 (14)	0.75471 (14)	0.0285 (4)	
N2	0.14744 (17)	0.18478 (14)	0.45096 (14)	0.0274 (4)	
C1	−0.0143 (2)	0.12842 (18)	0.84422 (17)	0.0275 (4)	
C2	−0.1057 (2)	0.0764 (2)	0.9223 (2)	0.0364 (5)	
C3	−0.2036 (2)	0.1445 (2)	0.99230 (19)	0.0379 (6)	
C4	−0.2109 (2)	0.2623 (2)	0.98686 (19)	0.0378 (6)	
C5	−0.1214 (2)	0.3150 (2)	0.91101 (19)	0.0367 (5)	
C6	−0.0223 (2)	0.24736 (17)	0.83812 (17)	0.0264 (4)	
C7	0.15016 (19)	0.20478 (16)	0.70011 (16)	0.0236 (4)	
C8	0.2599 (2)	0.22277 (17)	0.60006 (16)	0.0258 (4)	
C9	0.19052 (19)	0.25650 (16)	0.50325 (16)	0.0248 (4)	
C10	0.0816 (2)	0.24018 (17)	0.37034 (16)	0.0267 (4)	
C11	0.0191 (2)	0.1870 (2)	0.30272 (19)	0.0359 (5)	
C12	−0.0461 (2)	0.2534 (2)	0.2295 (2)	0.0428 (6)	
C13	−0.0488 (2)	0.3704 (2)	0.2221 (2)	0.0445 (6)	
C14	0.0126 (3)	0.4251 (2)	0.2877 (2)	0.0413 (6)	
C15	0.0782 (2)	0.35846 (18)	0.36298 (17)	0.0305 (5)	
C16	0.3328 (2)	0.3175 (2)	0.6252 (2)	0.0349 (5)	
C17	0.3583 (2)	0.1129 (2)	0.5719 (2)	0.0339 (5)	
S1'	0.50721 (6)	0.09629 (5)	0.85604 (5)	0.03416 (15)	
S2'	0.40874 (6)	0.43208 (4)	1.12466 (5)	0.03095 (15)	
N1'	0.53067 (17)	0.31085 (14)	0.83732 (14)	0.0276 (4)	
N2'	0.45605 (18)	0.21160 (15)	1.14371 (15)	0.0313 (4)	
C1'	0.6330 (2)	0.13756 (19)	0.75345 (17)	0.0324 (5)	
C2'	0.7266 (3)	0.0710 (2)	0.6744 (2)	0.0429 (6)	
C3'	0.8170 (3)	0.1245 (3)	0.6013 (2)	0.0497 (7)	
C4'	0.8160 (2)	0.2417 (3)	0.6045 (2)	0.0467 (7)	
C5'	0.7239 (2)	0.3082 (2)	0.68130 (19)	0.0389 (6)	
C6'	0.6309 (2)	0.25492 (18)	0.75653 (17)	0.0288 (5)	
C7'	0.4600 (2)	0.23898 (16)	0.89364 (16)	0.0245 (4)	
C8'	0.3426 (2)	0.27369 (17)	0.98818 (17)	0.0268 (4)	
C9'	0.4025 (2)	0.29371 (16)	1.08699 (17)	0.0256 (4)	
C10'	0.5093 (2)	0.25520 (18)	1.22437 (17)	0.0292 (5)	
C11'	0.5780 (3)	0.1898 (2)	1.2976 (2)	0.0395 (6)	
C12'	0.6284 (2)	0.2443 (2)	1.3717 (2)	0.0426 (6)	
C13'	0.6111 (2)	0.3625 (2)	1.37387 (19)	0.0390 (6)	
C14'	0.5442 (2)	0.4291 (2)	1.30219 (19)	0.0361 (5)	
C15'	0.4932 (2)	0.37455 (17)	1.22706 (17)	0.0278 (5)	
C16'	0.2577 (2)	0.3819 (2)	0.9530 (2)	0.0331 (5)	
C17'	0.2573 (3)	0.1780 (2)	1.0184 (2)	0.0368 (5)	
H2	−0.099 (2)	−0.002 (2)	0.925 (2)	0.049 (7)*	
H2'	0.727 (2)	−0.010 (2)	0.6737 (18)	0.036 (6)*	
H3	−0.269 (2)	0.1105 (19)	1.045 (2)	0.042 (7)*	
H3'	0.882 (3)	0.081 (2)	0.553 (2)	0.050 (7)*	
H4	−0.275 (2)	0.307 (2)	1.0317 (19)	0.039 (7)*	
H4'	0.874 (3)	0.278 (2)	0.551 (2)	0.066 (9)*	
H5	−0.127 (2)	0.398 (2)	0.904 (2)	0.051 (7)*	
H5'	0.721 (2)	0.3962 (19)	0.6852 (18)	0.038 (6)*	
H11	0.023 (2)	0.109 (2)	0.307 (2)	0.045 (7)*	
H11'	0.590 (3)	0.115 (2)	1.295 (2)	0.058 (8)*	
H12	−0.086 (3)	0.219 (2)	0.181 (2)	0.056 (8)*	
H12'	0.679 (2)	0.198 (2)	1.418 (2)	0.045 (7)*	
H13	−0.091 (2)	0.410 (2)	0.163 (2)	0.043 (7)*	
H13'	0.648 (2)	0.3967 (18)	1.4283 (18)	0.033 (6)*	
H14	0.009 (2)	0.5014 (19)	0.2881 (18)	0.034 (6)*	
H14'	0.531 (2)	0.5098 (19)	1.3029 (18)	0.033 (6)*	
H16A	0.373 (2)	0.2928 (19)	0.687 (2)	0.041 (7)*	
H16B	0.398 (2)	0.3325 (19)	0.5604 (19)	0.039 (6)*	
H16C	0.265 (2)	0.3889 (19)	0.6484 (18)	0.036 (6)*	
H16D	0.184 (2)	0.4074 (18)	1.0135 (19)	0.036 (6)*	
H16E	0.223 (2)	0.3664 (18)	0.8903 (19)	0.033 (6)*	
H16F	0.309 (2)	0.442 (2)	0.9308 (18)	0.033 (6)*	
H17A	0.425 (2)	0.1270 (19)	0.508 (2)	0.042 (7)*	
H17B	0.397 (2)	0.093 (2)	0.633 (2)	0.039 (7)*	
H17C	0.316 (2)	0.052 (2)	0.5537 (19)	0.037 (6)*	
H17D	0.224 (3)	0.163 (2)	0.954 (2)	0.060 (8)*	
H17E	0.187 (3)	0.203 (2)	1.079 (2)	0.056 (8)*	
H17F	0.308 (2)	0.110 (2)	1.0472 (19)	0.040 (7)*	

### Atomic displacement parameters (Å^2^)

**Table:**

	*U*^11^	*U*^22^	*U*^33^	*U*^12^	*U*^13^	*U*^23^
S1	0.0344 (3)	0.0212 (3)	0.0415 (3)	−0.0073 (2)	−0.0008 (2)	−0.0024 (2)
S2	0.0574 (4)	0.0217 (3)	0.0366 (3)	−0.0094 (3)	−0.0153 (3)	0.0029 (2)
N1	0.0336 (10)	0.0234 (9)	0.0275 (9)	−0.0048 (8)	−0.0037 (8)	0.0008 (7)
N2	0.0304 (9)	0.0253 (9)	0.0269 (9)	−0.0047 (7)	−0.0063 (8)	−0.0014 (7)
C1	0.0280 (11)	0.0306 (11)	0.0267 (11)	−0.0098 (9)	−0.0076 (9)	−0.0009 (9)
C2	0.0367 (13)	0.0360 (13)	0.0384 (13)	−0.0158 (11)	−0.0059 (10)	0.0055 (10)
C3	0.0339 (13)	0.0538 (15)	0.0284 (12)	−0.0171 (11)	−0.0057 (10)	0.0058 (11)
C4	0.0321 (12)	0.0517 (15)	0.0264 (12)	−0.0020 (11)	−0.0008 (10)	−0.0041 (11)
C5	0.0416 (13)	0.0331 (13)	0.0320 (13)	0.0000 (11)	−0.0038 (10)	−0.0021 (10)
C6	0.0275 (10)	0.0282 (11)	0.0248 (11)	−0.0043 (9)	−0.0084 (9)	0.0018 (8)
C7	0.0253 (10)	0.0234 (10)	0.0247 (10)	−0.0061 (8)	−0.0096 (8)	0.0007 (8)
C8	0.0279 (10)	0.0262 (10)	0.0244 (10)	−0.0070 (8)	−0.0062 (8)	0.0011 (8)
C9	0.0254 (10)	0.0217 (10)	0.0257 (11)	−0.0045 (8)	−0.0005 (8)	−0.0006 (8)
C10	0.0254 (10)	0.0289 (11)	0.0236 (11)	−0.0016 (9)	−0.0016 (8)	−0.0004 (8)
C11	0.0379 (13)	0.0385 (14)	0.0334 (13)	−0.0075 (11)	−0.0103 (10)	−0.0019 (10)
C12	0.0371 (13)	0.0606 (17)	0.0308 (13)	−0.0027 (12)	−0.0104 (11)	−0.0032 (12)
C13	0.0416 (14)	0.0590 (17)	0.0273 (13)	0.0082 (12)	−0.0081 (11)	0.0056 (12)
C14	0.0524 (15)	0.0316 (13)	0.0340 (13)	0.0044 (12)	−0.0054 (11)	0.0066 (10)
C15	0.0357 (12)	0.0299 (11)	0.0230 (11)	−0.0012 (9)	−0.0025 (9)	0.0009 (9)
C16	0.0353 (13)	0.0420 (14)	0.0313 (13)	−0.0176 (11)	−0.0064 (11)	−0.0010 (11)
C17	0.0293 (12)	0.0379 (13)	0.0324 (13)	0.0011 (10)	−0.0064 (10)	−0.0001 (10)
S1'	0.0426 (3)	0.0233 (3)	0.0359 (3)	−0.0007 (2)	−0.0079 (3)	−0.0062 (2)
S2'	0.0405 (3)	0.0226 (3)	0.0323 (3)	−0.0048 (2)	−0.0129 (2)	−0.0014 (2)
N1'	0.0282 (9)	0.0284 (9)	0.0259 (9)	−0.0047 (7)	−0.0033 (7)	−0.0048 (7)
N2'	0.0377 (10)	0.0258 (9)	0.0294 (10)	−0.0036 (8)	−0.0050 (8)	−0.0016 (7)
C1'	0.0316 (11)	0.0379 (12)	0.0272 (11)	0.0067 (10)	−0.0119 (9)	−0.0083 (9)
C2'	0.0430 (14)	0.0448 (15)	0.0385 (14)	0.0138 (12)	−0.0151 (12)	−0.0137 (12)
C3'	0.0353 (14)	0.078 (2)	0.0301 (14)	0.0172 (14)	−0.0085 (11)	−0.0159 (13)
C4'	0.0315 (13)	0.075 (2)	0.0299 (14)	−0.0016 (13)	−0.0028 (11)	−0.0033 (13)
C5'	0.0322 (12)	0.0533 (16)	0.0306 (12)	−0.0082 (11)	−0.0039 (10)	−0.0007 (11)
C6'	0.0263 (11)	0.0368 (12)	0.0241 (11)	−0.0021 (9)	−0.0074 (9)	−0.0047 (9)
C7'	0.0276 (10)	0.0211 (10)	0.0263 (11)	−0.0025 (8)	−0.0095 (8)	−0.0026 (8)
C8'	0.0280 (11)	0.0259 (11)	0.0260 (11)	−0.0054 (9)	−0.0026 (9)	−0.0032 (8)
C9'	0.0277 (10)	0.0218 (10)	0.0264 (11)	−0.0065 (8)	−0.0012 (8)	−0.0005 (8)
C10'	0.0300 (11)	0.0296 (11)	0.0265 (11)	−0.0052 (9)	−0.0021 (9)	0.0010 (9)
C11'	0.0457 (14)	0.0354 (14)	0.0360 (13)	−0.0018 (11)	−0.0107 (11)	0.0077 (11)
C12'	0.0401 (14)	0.0534 (16)	0.0347 (13)	−0.0059 (12)	−0.0127 (11)	0.0108 (12)
C13'	0.0375 (13)	0.0541 (16)	0.0294 (13)	−0.0155 (12)	−0.0093 (10)	−0.0007 (11)
C14'	0.0376 (13)	0.0373 (13)	0.0361 (13)	−0.0103 (11)	−0.0095 (10)	−0.0021 (10)
C15'	0.0280 (11)	0.0289 (11)	0.0255 (11)	−0.0050 (9)	−0.0029 (9)	0.0014 (9)
C16'	0.0328 (12)	0.0328 (12)	0.0327 (13)	0.0005 (10)	−0.0074 (10)	−0.0054 (10)
C17'	0.0354 (13)	0.0388 (14)	0.0380 (14)	−0.0152 (11)	−0.0029 (11)	−0.0037 (11)

### Geometric parameters (Å, °)

**Table:**

S1—C1	1.726 (2)	S1'—C1'	1.731 (2)
S1—C7	1.7455 (19)	S1'—C7'	1.749 (2)
S2—C15	1.729 (2)	S2'—C15'	1.732 (2)
S2—C9	1.752 (2)	S2'—C9'	1.749 (2)
N1—C7	1.288 (2)	N1'—C7'	1.293 (3)
N1—C6	1.398 (3)	N1'—C6'	1.393 (3)
N2—C9	1.283 (2)	N2'—C9'	1.289 (3)
N2—C10	1.398 (3)	N2'—C10'	1.394 (3)
C1—C2	1.397 (3)	C1'—C6'	1.394 (3)
C1—C6	1.401 (3)	C1'—C2'	1.403 (3)
C2—C3	1.373 (3)	C2'—C3'	1.369 (4)
C2—H2	0.92 (2)	C2'—H2'	0.96 (2)
C3—C4	1.388 (3)	C3'—C4'	1.394 (4)
C3—H3	0.96 (2)	C3'—H3'	0.91 (3)
C4—C5	1.375 (3)	C4'—C5'	1.381 (3)
C4—H4	0.89 (2)	C4'—H4'	0.93 (3)
C5—C6	1.396 (3)	C5'—C6'	1.397 (3)
C5—H5	0.98 (2)	C5'—H5'	1.04 (2)
C7—C8	1.527 (3)	C7'—C8'	1.524 (3)
C8—C9	1.522 (3)	C8'—C9'	1.524 (3)
C8—C16	1.531 (3)	C8'—C16'	1.534 (3)
C8—C17	1.532 (3)	C8'—C17'	1.534 (3)
C10—C11	1.390 (3)	C10'—C11'	1.393 (3)
C10—C15	1.399 (3)	C10'—C15'	1.403 (3)
C11—C12	1.378 (3)	C11'—C12'	1.379 (3)
C11—H11	0.92 (2)	C11'—H11'	0.88 (3)
C12—C13	1.384 (4)	C12'—C13'	1.388 (4)
C12—H12	0.94 (3)	C12'—H12'	0.95 (2)
C13—C14	1.374 (4)	C13'—C14'	1.375 (3)
C13—H13	0.99 (2)	C13'—H13'	0.97 (2)
C14—C15	1.400 (3)	C14'—C15'	1.394 (3)
C14—H14	0.90 (2)	C14'—H14'	0.95 (2)
C16—H16A	0.95 (2)	C16'—H16D	0.97 (2)
C16—H16B	0.96 (2)	C16'—H16E	0.95 (2)
C16—H16C	1.02 (2)	C16'—H16F	0.95 (2)
C17—H17A	0.96 (3)	C17'—H17D	0.97 (3)
C17—H17B	0.92 (2)	C17'—H17E	0.95 (3)
C17—H17C	0.96 (2)	C17'—H17F	0.98 (2)
			
C1—S1—C7	89.31 (10)	C1'—S1'—C7'	88.76 (10)
C15—S2—C9	89.11 (10)	C15'—S2'—C9'	88.97 (10)
C7—N1—C6	111.00 (17)	C7'—N1'—C6'	110.43 (17)
C9—N2—C10	110.91 (17)	C9'—N2'—C10'	110.13 (18)
C2—C1—C6	120.6 (2)	C6'—C1'—C2'	121.1 (2)
C2—C1—S1	129.91 (18)	C6'—C1'—S1'	109.69 (16)
C6—C1—S1	109.51 (15)	C2'—C1'—S1'	129.2 (2)
C3—C2—C1	118.5 (2)	C3'—C2'—C1'	118.0 (3)
C3—C2—H2	122.4 (16)	C3'—C2'—H2'	122.1 (14)
C1—C2—H2	119.2 (16)	C1'—C2'—H2'	119.8 (14)
C2—C3—C4	121.0 (2)	C2'—C3'—C4'	121.2 (2)
C2—C3—H3	119.6 (14)	C2'—C3'—H3'	118.8 (17)
C4—C3—H3	119.4 (14)	C4'—C3'—H3'	119.9 (17)
C5—C4—C3	121.4 (2)	C5'—C4'—C3'	121.3 (3)
C5—C4—H4	117.6 (15)	C5'—C4'—H4'	118.2 (18)
C3—C4—H4	121.0 (15)	C3'—C4'—H4'	120.3 (18)
C4—C5—C6	118.5 (2)	C4'—C5'—C6'	118.2 (2)
C4—C5—H5	122.3 (15)	C4'—C5'—H5'	122.8 (13)
C6—C5—H5	119.1 (15)	C6'—C5'—H5'	119.0 (13)
C5—C6—N1	125.49 (19)	N1'—C6'—C1'	115.01 (19)
C5—C6—C1	120.1 (2)	N1'—C6'—C5'	124.8 (2)
N1—C6—C1	114.43 (18)	C1'—C6'—C5'	120.2 (2)
N1—C7—C8	122.99 (17)	N1'—C7'—C8'	123.16 (18)
N1—C7—S1	115.73 (15)	N1'—C7'—S1'	116.10 (15)
C8—C7—S1	121.24 (14)	C8'—C7'—S1'	120.74 (14)
C9—C8—C7	106.21 (15)	C7'—C8'—C9'	106.10 (16)
C9—C8—C16	111.35 (18)	C7'—C8'—C16'	108.99 (17)
C7—C8—C16	108.85 (17)	C9'—C8'—C16'	111.91 (17)
C9—C8—C17	108.69 (17)	C7'—C8'—C17'	110.90 (18)
C7—C8—C17	111.44 (17)	C9'—C8'—C17'	109.02 (18)
C16—C8—C17	110.24 (19)	C16'—C8'—C17'	109.87 (19)
N2—C9—C8	123.26 (18)	N2'—C9'—C8'	122.76 (18)
N2—C9—S2	115.81 (15)	N2'—C9'—S2'	116.46 (16)
C8—C9—S2	120.88 (14)	C8'—C9'—S2'	120.71 (14)
C11—C10—N2	125.01 (19)	C11'—C10'—N2'	125.0 (2)
C11—C10—C15	120.2 (2)	C11'—C10'—C15'	119.6 (2)
N2—C10—C15	114.78 (18)	N2'—C10'—C15'	115.36 (18)
C12—C11—C10	118.5 (2)	C12'—C11'—C10'	119.0 (2)
C12—C11—H11	121.8 (16)	C12'—C11'—H11'	120.9 (18)
C10—C11—H11	119.7 (16)	C10'—C11'—H11'	120.1 (18)
C11—C12—C13	121.2 (2)	C11'—C12'—C13'	120.9 (2)
C11—C12—H12	119.6 (17)	C11'—C12'—H12'	117.4 (15)
C13—C12—H12	119.2 (16)	C13'—C12'—H12'	121.6 (15)
C14—C13—C12	121.5 (2)	C14'—C13'—C12'	121.4 (2)
C14—C13—H13	122.9 (14)	C14'—C13'—H13'	121.0 (13)
C12—C13—H13	115.3 (14)	C12'—C13'—H13'	117.7 (13)
C13—C14—C15	117.8 (2)	C13'—C14'—C15'	118.0 (2)
C13—C14—H14	124.2 (14)	C13'—C14'—H14'	122.6 (13)
C15—C14—H14	117.9 (15)	C15'—C14'—H14'	119.4 (13)
C10—C15—C14	120.9 (2)	C14'—C15'—C10'	121.2 (2)
C10—C15—S2	109.38 (15)	C14'—C15'—S2'	129.73 (17)
C14—C15—S2	129.72 (19)	C10'—C15'—S2'	109.08 (15)
C8—C16—H16A	107.9 (14)	C8'—C16'—H16D	110.2 (13)
C8—C16—H16B	109.1 (13)	C8'—C16'—H16E	109.4 (13)
H16A—C16—H16B	112.0 (19)	H16D—C16'—H16E	109.8 (18)
C8—C16—H16C	109.6 (12)	C8'—C16'—H16F	111.6 (13)
H16A—C16—H16C	106.8 (18)	H16D—C16'—H16F	109.2 (18)
H16B—C16—H16C	111.4 (18)	H16E—C16'—H16F	106.7 (18)
C8—C17—H17A	109.2 (14)	C8'—C17'—H17D	108.2 (16)
C8—C17—H17B	106.9 (15)	C8'—C17'—H17E	107.9 (16)
H17A—C17—H17B	111 (2)	H17D—C17'—H17E	112 (2)
C8—C17—H17C	112.3 (14)	C8'—C17'—H17F	111.2 (13)
H17A—C17—H17C	106.9 (19)	H17D—C17'—H17F	113 (2)
H17B—C17—H17C	111 (2)	H17E—C17'—H17F	105 (2)
			
C7—S1—C1—C2	178.3 (2)	C7'—S1'—C1'—C6'	0.84 (15)
C7—S1—C1—C6	−0.96 (15)	C7'—S1'—C1'—C2'	−178.3 (2)
C6—C1—C2—C3	0.3 (3)	C6'—C1'—C2'—C3'	0.9 (3)
S1—C1—C2—C3	−178.91 (17)	S1'—C1'—C2'—C3'	179.97 (18)
C1—C2—C3—C4	−0.8 (4)	C1'—C2'—C3'—C4'	−0.4 (4)
C2—C3—C4—C5	0.4 (4)	C2'—C3'—C4'—C5'	0.0 (4)
C3—C4—C5—C6	0.5 (4)	C3'—C4'—C5'—C6'	0.0 (4)
C4—C5—C6—N1	178.3 (2)	C7'—N1'—C6'—C1'	0.3 (2)
C4—C5—C6—C1	−0.9 (3)	C7'—N1'—C6'—C5'	179.6 (2)
C7—N1—C6—C5	−179.0 (2)	C2'—C1'—C6'—N1'	178.42 (19)
C7—N1—C6—C1	0.2 (2)	S1'—C1'—C6'—N1'	−0.8 (2)
C2—C1—C6—C5	0.6 (3)	C2'—C1'—C6'—C5'	−0.9 (3)
S1—C1—C6—C5	179.95 (17)	S1'—C1'—C6'—C5'	179.83 (16)
C2—C1—C6—N1	−178.73 (19)	C4'—C5'—C6'—N1'	−178.8 (2)
S1—C1—C6—N1	0.7 (2)	C4'—C5'—C6'—C1'	0.4 (3)
C6—N1—C7—C8	176.71 (17)	C6'—N1'—C7'—C8'	179.98 (17)
C6—N1—C7—S1	−1.0 (2)	C6'—N1'—C7'—S1'	0.4 (2)
C1—S1—C7—N1	1.18 (16)	C1'—S1'—C7'—N1'	−0.74 (17)
C1—S1—C7—C8	−176.57 (16)	C1'—S1'—C7'—C8'	179.65 (16)
N1—C7—C8—C9	−77.6 (2)	N1'—C7'—C8'—C9'	−76.5 (2)
S1—C7—C8—C9	99.98 (17)	S1'—C7'—C8'—C9'	103.05 (17)
N1—C7—C8—C16	42.4 (3)	N1'—C7'—C8'—C16'	44.1 (3)
S1—C7—C8—C16	−140.02 (16)	S1'—C7'—C8'—C16'	−136.29 (16)
N1—C7—C8—C17	164.18 (19)	N1'—C7'—C8'—C17'	165.2 (2)
S1—C7—C8—C17	−18.2 (2)	S1'—C7'—C8'—C17'	−15.2 (2)
C10—N2—C9—C8	176.62 (17)	C10'—N2'—C9'—C8'	176.41 (17)
C10—N2—C9—S2	−0.8 (2)	C10'—N2'—C9'—S2'	−0.5 (2)
C7—C8—C9—N2	−77.1 (2)	C7'—C8'—C9'—N2'	−74.6 (2)
C16—C8—C9—N2	164.55 (19)	C16'—C8'—C9'—N2'	166.66 (19)
C17—C8—C9—N2	42.9 (3)	C17'—C8'—C9'—N2'	44.9 (3)
C7—C8—C9—S2	100.20 (17)	C7'—C8'—C9'—S2'	102.22 (17)
C16—C8—C9—S2	−18.2 (2)	C16'—C8'—C9'—S2'	−16.5 (2)
C17—C8—C9—S2	−139.79 (16)	C17'—C8'—C9'—S2'	−138.29 (17)
C15—S2—C9—N2	0.49 (17)	C15'—S2'—C9'—N2'	0.39 (17)
C15—S2—C9—C8	−176.98 (16)	C15'—S2'—C9'—C8'	−176.60 (16)
C9—N2—C10—C11	−177.3 (2)	C9'—N2'—C10'—C11'	−177.9 (2)
C9—N2—C10—C15	0.8 (2)	C9'—N2'—C10'—C15'	0.4 (3)
N2—C10—C11—C12	177.7 (2)	N2'—C10'—C11'—C12'	178.4 (2)
C15—C10—C11—C12	−0.3 (3)	C15'—C10'—C11'—C12'	0.2 (3)
C10—C11—C12—C13	0.6 (4)	C10'—C11'—C12'—C13'	0.1 (4)
C11—C12—C13—C14	−0.4 (4)	C11'—C12'—C13'—C14'	−0.4 (4)
C12—C13—C14—C15	−0.1 (4)	C12'—C13'—C14'—C15'	0.3 (4)
C11—C10—C15—C14	−0.2 (3)	C13'—C14'—C15'—C10'	0.0 (3)
N2—C10—C15—C14	−178.41 (19)	C13'—C14'—C15'—S2'	−178.20 (17)
C11—C10—C15—S2	177.81 (17)	C11'—C10'—C15'—C14'	−0.3 (3)
N2—C10—C15—S2	−0.4 (2)	N2'—C10'—C15'—C14'	−178.64 (19)
C13—C14—C15—C10	0.4 (3)	C11'—C10'—C15'—S2'	178.25 (17)
C13—C14—C15—S2	−177.15 (18)	N2'—C10'—C15'—S2'	−0.1 (2)
C9—S2—C15—C10	−0.03 (16)	C9'—S2'—C15'—C14'	178.2 (2)
C9—S2—C15—C14	177.8 (2)	C9'—S2'—C15'—C10'	−0.14 (16)
